# Clinical and Non‐clinical Evidence Supporting the Development of Masupirdine (SUVN‐502) for the Management of Agitation in Alzheimer’s Disease

**DOI:** 10.1002/alz70859_099716

**Published:** 2025-12-25

**Authors:** Ramakrishna Nirogi, Renny Abraham, Jyothsna Ravulu, Satish Jetta, Vijay Benade, Anil Shinde, Abdul Rasheed Mohammed, Vinod Kumar Goyal, Veera Raghava Chowdary Palacharla, Ramkumar Subramanian, Pradeep Jayarajan

**Affiliations:** ^1^ Suven Life Sciences Ltd, Hyderabad, Telangana India

## Abstract

**Background:**

Neuropsychiatric symptoms (NPS) commonly occur over the course of Alzheimer's disease (AD), with agitation being one of the most prevalent symptoms. Agitation results in caregiver distress, increased morbidity and mortality, and early institutionalization of patients with AD. Pharmacological agents used to manage agitation are limited to antipsychotics, benzodiazepines, antiepileptics, and antidepressants, for which efficacy and safety are not well established, except for brexpiprazole. The side effects associated with these agents are substantial, highlighting the need for effective and safe treatments.

Serotonin‐6 (5‐HT_6_) receptors are widely expressed in the brain regions involved in control of mood and behavior. Modulation of 5‐HT_6_ receptors may have potential therapeutic implications in the management of agitation in AD.

**Method:**

Masupirdine, a pure, potent and selective 5‐HT_6_ receptor antagonist was assessed for its effects on aggression like behaviors in rodent models like resident‐intruder task and dominant‐submissive assay. The effects of Masupirdine on cognitive and motor performances were studied in the alternating lever cyclic ratio schedule. Clinical evidence supporting the potential effectiveness of Masupirdine for treating agitation was generated from a subgroup analysis of a Phase‐2, 26‐week proof‐of‐concept clinical study in AD patients (NCT02580305).

**Result:**

Oral treatment with Masupirdine led to a significant and meaningful reduction in aggressive behaviors, as observed in both the resident‐intruder and dominant‐submissive assays. Performance on the alternating lever cyclic ration schedule demonstrated that Masupirdine did not induce any cognitive or motor impairments. Subgroup analysis of the agitation/aggression domains on the NPI‐12 scale revealed a statistically significant reduction in agitation/aggression scores (p<0.01) in patients treated with either 50 or 100 mg of Masupirdine at Week 13, with the effect remaining sustained through the end of 26‐week treatment period.

**Conclusion:**

The non‐clinical and clinical data support further evaluation of Masupirdine for the management of agitation in AD. A global Phase‐3, double‐blind, randomized, placebo‐controlled study to establish the efficacy, safety, tolerability, and pharmacokinetics of Masupirdine in patients with agitation in dementia of the Alzheimer’s type (NCT05397639 and EudraCT 2021‐003405‐22) is in progress.

